# The promising prognostic value of vagal nerve activity at the initial management of ovarian cancer

**DOI:** 10.3389/fonc.2022.1049970

**Published:** 2022-11-29

**Authors:** François Cherifi, Sophie Lefevre Arbogast, Jonaz Font, Cyril Abdeddaim, Stephanie Becourt, Nicolas Penel, Elodie Coquan, Justine Lequesne, Yori Gidron, Florence Joly

**Affiliations:** ^1^ Department of Medical Oncology, Centre François Baclesse, Caen, France; ^2^ Department of Clinical Research, Centre Francois Baclesse, Caen, France; ^3^ Normandie Université, UNICAEN, Institut National de la Santé et de la Recherche Médicale (Inserm) U1086, ANTICIPE, Caen, France; ^4^ Department of Cardiology, Centre Hospitalier Universitaire de Caen, Caen, France; ^5^ Department of Medical Oncology, Centre Oscar Lambret, Lille, France; ^6^ Department of Nursing, Faculty of Health Sciences, Haifa University, Haifa, Israel

**Keywords:** vagus nerve, ovarian cancer, heart rate variability (HRV), autonomic nervous system, prognostic factor and survival, parasympathetic activity

## Abstract

**Objective:**

Identifying new modifiable prognostic markers is important for ovarian cancer (OC). Low parasympathic activity is associated with inflammation, oxidative stress and sympathetic nervous system activation. Previous studies reported that low vagal nerve activity, measured by low heart rate variability (HRV), may predict poor cancer prognosis. We aimed to examine the prognostic value of HRV in OC.

**Methods:**

This bicentric retrospective study included patients diagnosed with serous OC FIGO stage ≥IIB, between January 2015 and August 2019, with electrocardiograms (ECG) available around diagnosis. HRV was measured from ECG using the time domain parameter of standard deviation of all normal-to-normal heartbeat intervals (SDNN). Optimal SDNN cut-off was determined using the Youden index criteria of time-dependent ROC curves. We used multivariate cox proportional hazard models to investigate the association between HRV and overall survival (OS), while adjusting for well-known OC prognostic factors.

**Results:**

The 202 patients included were 65.7 years-old on average, 93% had stage FIGO IIIC/IV, 56% had complete surgical resection. Median OS was 38.6 months [95%CI:34.4-47.4]. The median SDNN was 11.1ms, with an optimal cut-off of 10ms to predict OS. OS was shorter for patients with low HRV compared to high HRV (26.4 vs 45.1 months; p<0.001). In multivariate analysis, HRV remained an independent prognostic factor with a two-fold higher risk of death among patients with low SDNN compared to those with high SDNN (HR=2.03, 95%CI=1.35-3.06, p<0.001).

**Conclusion:**

Low HRV, was associated with worse OS in OC patients, supporting previous studies on the prognostic role of HRV in cancer. If replicated in prospective studies, vagal nerve activity may be a new therapeutic target in OC.

## Introduction

The management of ovarian cancer (OC) has evolved with improvements in the techniques and indications of surgery and with the recent development of new treatments, such as PARP or VEGF inhibitors (1). However, the 5 years survival remains poor at approximately 44% (1). Although stage, residual disease after surgery, breast cancer gene (BRCA) 1 and 2 mutations, and levels of the serum marker cancer antigen 125 (CA125) are known prognostic factors for OC survival, there is a need to identify new prognostic factors to better target patients requiring more aggressive treatment or increased monitoring (1, 2). The ultimate objective is to identify new independent prognostic factors that could be modified by specific actions.

Inflammation and immune responses play a determining role in the different stages of tumor development (initiation, promotion, angiogenesis, invasion, and metastasis) (3, 4). In OC, the pro-inflammatory tumor microenvironment contributes to metastasis and chemoresistance (3). In addition, the nervous system has recently emerged as a crucial facilitator of cancer growth in many types of cancers (5). The autonomic nervous system, particularly the parasympathetic branch, has been shown to be one of the systems which control inflammatory processes (6, 7). Activation of the sympathetic nervous system (SNS) plays a role in the initiation and progression of cancer through the activation of stress hormones such as catecholamines (8, 9). More than two-thirds of all patients with cancer present autonomic dysfunction (10). In contrast, the parasympathetic nervous system (PNS), which mainly involves the vagus nerve, reduces oxidative stress, inhibits inflammation, and inhibits sympathetic overactivity. The vagus inhibits inflammation through a reflex neuro-hormonal circuit by activating the hypothalamic-pituitary-adrenal axis, leading to cortisol secretion, and by activating sympathetic innervation of the spleen, leading to certain T-cells suppressing macrophages from synthesizing inflammatory cytokines (11, 12). Hence, high parasympathetic activity may result in antitumor activity (7, 11, 13).

Vagal nerve activity can be indexed in a non-invasive manner by measuring the heart rate variability (HRV) in electrocardiograms (ECG). HRV refers to fluctuations in the intervals between successive normal R-R intervals (the time between each QRS complex of an ECG) (14, 15). Given that parasympathetic nerve traffic regulates the heart-rate faster than sympathetic outflow, heart beat-to-beat changes are considered mostly a reflection of vagal activity (14, 16, 17). There is a strong correlation between lower HRV activity and morbidity/mortality in a certain number of diseases associated with excessive inflammation, including cancer (11). Some studies showed an association between a high HRV value and better survival rate in several cancers (e.g. breast, pancreas, prostate) (18–24). However, these studies were heterogeneous, and several had small sample sizes. Finally, none of the published studies focused on OC.

Higher HRV at baseline may also predict decreases in biological markers (e.g., tumor and inflammatory markers). For example, in colorectal cancer, high HRV has been associated with lower carcinoembryonic antigen (CEA) level other time (25). In pancreatic cancer, it was found that the association between initial HRV and survival was statistically mediated by inflammatory biomarkers namely C-reactive protein (23). Among the inflammatory prognostic factors, the neutrophil-to-lymphocyte ratio (NLR) is strongly associated with overall survival (OS) in OC (26, 27). However, the potential link between the NLR and vagal nerve activity, which modulates inflammation, remains unknown.

The primary objective of this study was to examine whether vagus nerve activity, indexed through the measurement of HRV, is an independent prognostic factor for OS during the initial management of ovarian cancer. The second objective was to explore the association of HRV with progression-free survival and other strong biological prognostic factors, such as CA125 and NLR.

## Materials and methods

### Study design and population

We conducted a retrospective (pseudo-prospective), bicentric observational study.

### Patient cohort

We exhaustively included all patients newly diagnosed and treated at the Comprehensive Cancer Center François Baclesse (Caen, France) or Oscar Lambret (Lille, France) between January 1, 2015, and August 31, 2019, for epithelial OC FIGO stage IIB-IV, for whom an ECG was available. We excluded patients who had cardiac comorbidities, rendering the intervals between each QRS complex uninterpretable (e.g., active atrial fibrillation), and patients who opposed the collection and processing of their data.

### Vagal nerve activity and heart rate variability

We collected the 10 second ECG used in clinical routine, closest to the time of diagnosis within 12 months of the diagnosis. All the ECGs were analyzed and interpreted by a confirmed cardiologist, and he also carried out the measurements of the R-R intervals. The ECG was deemed uninterpretable if the rhythm was not sinus or if the quality of the ECG was not sufficient for reliable analysis. Vagal nerve activity was measured by deriving time-domain parameters of HRV, according to a method previously described in other studies (15, 28). The time-domain parameters used in our study included the standard deviation of all normal RR intervals (SDNN) in milliseconds (ms) and the square root of the mean of the squared differences between adjacent normal RR intervals (RMSSD) in ms.

### Background

General patient characteristics (age, weight, performance status, tobacco use), disease-related information (histology, FIGO stage, BRCA status), and treatment details were collected from the patients’ medical records. A history of cardiovascular disease (ischemic/rhythmic heart disease, hypertension, stroke, autoimmune disease) and use of other treatments that could alter HRV (antiarrhythmics, beta-blockers, anti-inflammatory, or corticosteroid medications) were also noted.

### Outcomes

Overall survival (OS) was determined from the time of diagnosis to the last follow-up or death. Progression-free survival (PFS) was determined from the time of diagnosis to disease progression or the last evaluation.

CA125 data were collected near the end of chemotherapy (CT) (or approximately 6 months after diagnosis for those not treated by CT), with values less than or equal to 35 UI/ml considered as normal (2). We examined the evolution of CA 125 over time by taking the measurements of the initial CA125, at 3, 6, 9, and 12 months, and progression, removing the post-progression measurements if it had occurred before 12 months. The neutrophil-to-lymphocyte ratio (NLR) was calculated from neutrophil and lymphocyte counts in blood tests available at the time of the ECG, with NLR higher than 4 considered as high NLR (26, 27).

### Statistical analysis

Patients clinical characteristics were described by means, standard deviation, and range for quantitative variables and by frequency and proportions for categorical factors and compared between study centers using Student t-tests and chi-squared tests.

In the primary analysis, we investigated the association between SDNN and OS. We identified the optimal SDNN cut-off for OS using the Youden index criteria of the time-dependent ROC curve and computed survival curves stratified by SDNN categories (low vs. high) using the Kaplan-Meier method. Multivariate associations of SDNN with OS were then tested using Cox proportional hazards regression models, adjusted for study center, age, cancer stage, ECOG performance status, NLR, CA125, and the result of surgery (R0 if complete, R1 if subtotal, or no surgery). SDNN was primarily considered a categorical variable (low vs. high HRV) and was also studied as a continuous measure using log-transformation.

In secondary analyses, we studied the association between SDNN and PFS using similar univariate and multivariate Cox regression models. We also assessed the association between SDNN and both CA125 levels at the end of first-line treatment and NLR at the time of ECG using chi-squared tests.

In the sensitivity analyses, we repeated the analyses using the RMSSD as a measure of HRV.

### Statement of ethics

This study respects French regulations for medical research. The registration number in the Health Data Hub was N° F20201023132151. Informed consent was obtained from all participants.

## Results

### Patient characteristic and treatment

**Figure 1:**
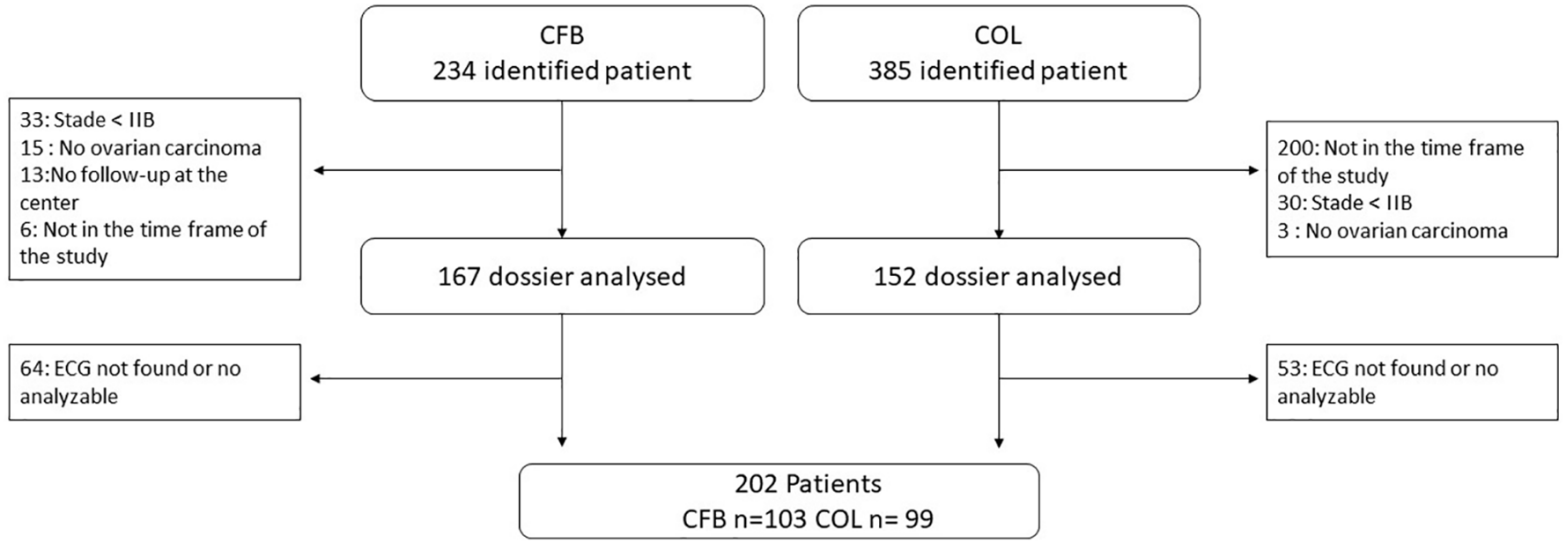
Flow chart. CFB, Francois Baclesse Center; Caen, COL; Oscar Lambret Center, Lille; ECG, Electrocardiograms.

In this study, 619 medical records of OC patients from two comprehensive cancer centers were identified (see **Figure 1** for flowchart). Among the 319 records of patients who met the inclusion criteria, the ECG was not available or analyzable for 117 patients. In total, 202 records were included in the final analysis.

### Patient description and treatment

The patient population is shown in **Table 1**. The median follow-up time was 31 months (range, 0.7 to 74.2). The mean age of the population was 65.7 (range: 20.0-87.0) years, and 173 (86%) had an ECOG score of 0/1. The majority of patients had high-grade serous OC (86%), with an advanced stage (93% with FIGO stage IIIC/IV). In addition, 17 patients (8%) had a cardiac history that included mostly resolved atrial fibrillation. Thirty-three patients (16%) were treated with beta-blockers at the time of ECG, in most cases, to manage high blood pressure. The recruitment was different between the two centers, with the inclusion of older and less performing patients in Caen.

**Table 1:** Patient and tumor characteristics of the global population and in each cancer center.

		Total (N=202)	CFB (N=103)	COL (N=99)	P-value
Age Mean (SD) (min-max)		65.7 (11.6) (20.0-87.0)	67.3 (11.8)	63.9 (11.3)	0.03480
ECOG Performance status	0	92 (46%)	38 (37%)	54 (55%)	**0.0104**
	1	81 (40%)	44 (43%)	37 (37%)	
	≥2	29 (14%)	21 (20%)	8 (8%)	
BMI Mean (SD) (min-max)		26.7 (6.06) (16.2-50.8)	26.7 (6.57)	26.8 (5.51)	0.871
Tabacco use	No	171 (85%)	97 (94%)	74 (75%)	**<0.001**
	Yes	17 (8%)	5 (5%)	12 (12%)	
	Weaned	14 (7%)	1 (1%)	13 (13%)	
History of heart disease	No	185 (92%)	92 (89%)	93 (94%)	0.353
	Yes	17 (8%)	11 (11%)	6 (6%)	
History of diabetes mellitus	No	178 (88%)	92 (89%)	86 (87%)	0.748
	Yes	24 (12%)	11 (11%)	13 (13%)	
History of HBP	No	113 (56%)	56 (54%)	57 (58%)	0.751
	Yes	89 (44%)	47 (46%)	42 (42%)	
History of stroke	No	194 (96%)	97 (94%)	97 (98%)	0.305
	Yes	8 (4%)	6 (6%)	2 (2%)	
History of DVT	No	184 (91%)	93 (90%)	91 (92%)	0.874
	Yes	18 (9%)	10 (10%)	8 (8%)	
History of autoimmune disease	No	187 (93%)	93 (90%)	94 (95%)	0.32
	Yes	15 (7%)	10 (10%)	5 (5%)	
Beta-blocker treatment	No	169(84%)	81 (79%)	82 (83%)	0.316
	Yes	33 (16%)	17 (17%)	16 (16%)	
Histology	HGS	174 (86%)	88 (85%)	86 (87%)	0.928
	Other	28 (14%)	15 (15%)	13 (13%)	
FIGO stage	IIB	4 (2%)	3 (3%)	1 (1%)	0.222
	IIC	1 (0%)	0 (0%)	1 (1%)	
	IIIA	3 (1%)	2 (2%)	1 (1%)	
	IIIB	7 (3%)	1 (1%)	6 (6%)	
	IIIC	136 (67%)	70 (68%)	66 (67%)	
	IVA	18 (9%)	12 (12%)	6 (6%)	
	IVB	33 (16%)	15 (15%)	18 (18%)	
BRCA mutational status	No muted	134 (66%)	74 (72%)	60 (61%)	0.126
	Muted	19 (9%)	6 (6%)	13 (13%)	
	Missing	49 (24%)	23 (22%)	26 (26%)	
Surgery	Primary surgery	43 (21%)	20 (19%)	23 (23%)	**<0.001**
	Interval surgery	58 (29%)	35 (34%)	23 (23%)	
	Terminal Surgery	39 (19%)	7 (7%)	32 (32%)	
	No surgery	62 (31%)	41 (40%)	21 (21%)	
R0 ressection	No	88 (44%)	57 (55%)	31 (31%)	**<0.001**
	Yes	114 (56%)	46 (45%)	68 (69%)	
Chemotherapy treatment	Adjuvant	42 (21%)	20 (20%)	22 (22%)	0.227
	Neo Adjuvant	103 (51%)	47 (46%)	56 (57%)	
	CT only	48 (24%)	30 (29%)	18 (18%)	
	No CT	8 (4%)	5 (5%)	3 (3%)	
	Missing	1 (0.5%)	1 (1.0%)	0 (0%)	
Bevacizumab maintenance	No	86 (43%)	45 (44%)	41 (42%)	0.855
	Yes	114 (57%)	57 (56%)	57 (58%)	
	Missing	2 (1.0%)	1 (1.0%)	1 (1.0%)	
Resumption of treatment after first progression	No	14 (9%)	7 (8%)	7 (10%)	0.961
	Yes	141 (91%)	77 (92%)	64 (90%)	
	Missing	47 (23.3%)	19 (18.4%)	28 (28.3%)	
Number of line Mean (SD) (min-max)		2.55 (1.55) (0-8)	2.59 (1.44)(0-6)	2.50 (1.66)(0-8)	0.684
PARPi use	No	150 (74%)	78 (75%)	72 (73%)	0.558
	Yes	52 (26%)	25 (24%)	27 (27%)	

ECOG, Eastern Cooperative Oncology Group; BMI, body mass index; CFB, Centre François Baclesse Caen France; COL, Centre Oscar Lambret Lille France; CT, Chemotherapy; DVT, deep vein thrombosis; HBP, high blood pressure; HGS, High grade Serous; PARPi, Poly (ADP-ribose) polymerase inhibitor; R0, Complete surgery; Standard Deviation. Bold values are significant value.

Almost all patients (96%) received chemotherapy with carboplatin and paclitaxel as the first-line treatment, and 114 (57%) received maintenance bevacizumab. One hundred and forty patients (69%) underwent surgery either first or after neoadjuvant chemotherapy. In total, 114 patients (56%) benefited from R0 resection (complete surgery). The median number of chemotherapy treatment lines after the initial surgery was 2.5 (0–8), with 93 patients (46%) receiving ≥3 lines.

The NLR was available for 95% of patients, with a median of 3.5 (IQR:2.1,5.7; range: 0.05,88.7). Seventy-six patients (40%) had high NLR (>4). The median CA 125 (measured the closest to the end of first line treatment) was 34.4 UI/ml (IQR:11.9,128.0; range: 2,9760).

The median time between the date of ECG and diagnosis was 2 days prior to diagnosis (IQR: -10, 107; range: -196, 368), and 136 (97%) were performed before surgery.

### HRV analysis

The median SDNN was 11.1 ms (min=1.93; max=74.5 ms) and RMSSD was 11.5 ms (min=1.70; max=84.8 ms). Using the Youden index, we found an optimal SDNN cut-off for OS at 10 ms, differentiating high from low HRV, and we applied the same cut-off for RMSDD due to similar results. Eighty patients (40%) were categorized as having low SDNN, and 122 (60%) as having high SDNN. There were no statistically significant differences in age, ECOG status, stage, diabetes history, beta-blocker treatment, number of surgeries, or R0 resection between the two HRV groups (**Table 2**). A total of 123 ECG were collected before chemotherapy (63%), without statistical differences between the two groups (p=0.6).

**Table 2:** Patient and tumor characteristics between low and high HRV.

		Low n=80	High n=122	P-value
Age Mean (SD)		67.4 (9.33)	64.5 (12.9)	0.0747
ECOG Performance status	0	32 (40%)	60 (49%)	0.256
	> 0	48 (60%)	62 (51%)	
Center	Lille	39 (49%)	64 (52%)	0.71
	Caen	41 (51%)	58 (48%)	
BMI Mean (SD)		27.7 (6.27)	26.1 (5.85)	0.0686
History of heart disease	No	73 (91%)	112 (92%)	1
	Yes	7 (9%)	10 (8%)	
History of diabetes mellitus	No	66 (82%)	112 (92%)	0.0757
	Yes	14 (18%)	10 (8%)	
Beta-blocker treatment	No	61 (76%)	108 (88%)	0.0818
	Yes	19 (24%)	14 (12%)	
Histology	HGS	69 (86%)	105 (86%)	1
	Other	11 (14%)	17 (14%)	
FIGO stage	<IV	56 (70%)	95 (78%)	0.274
	IV	24 (30%)	27 (22%)	
BRCA mutational status	No muted	45 (56%)	89 (73%)	0.00553
	Muted	6 (8%)	6 (8%)	
	Missing	29 (36%)	20 (16%)	
Surgery	Yes	52 (65%)	88 (72%)	0.358
	No	28 (35%)	34 (28%)	
R0 resection	No	38 (48%)	50 (41%)	0.442
	Yes	42 (52%)	72 (59%)	
NLR	≤ 4	40 (53%)	76 (65%)	0.145
	>4	35 (47%)	41 (35%)	
	Missing	5 (6.3%)	5 (4.1%)	
CA 125*	Normal	38 (48%)	66 (54%)	0.439
	High	42 (52%)	56 (46%)	

ECOG: Eastern Cooperative Oncology Group; BMI: body mass index; BRCA: Breast Cancer gene;HGS: High grade Serous; NLR: Neutrophil-to-lymphocyte ratio; R0: Complete surgery; SD: Standard Deviation; *: near the end of chemotherapy (CT) (or approximately 6 months after diagnosis for those not treated by CT

### Relationship between HRV and OS/PFS

#### Univariate analysis of OS

**Figure 2:**
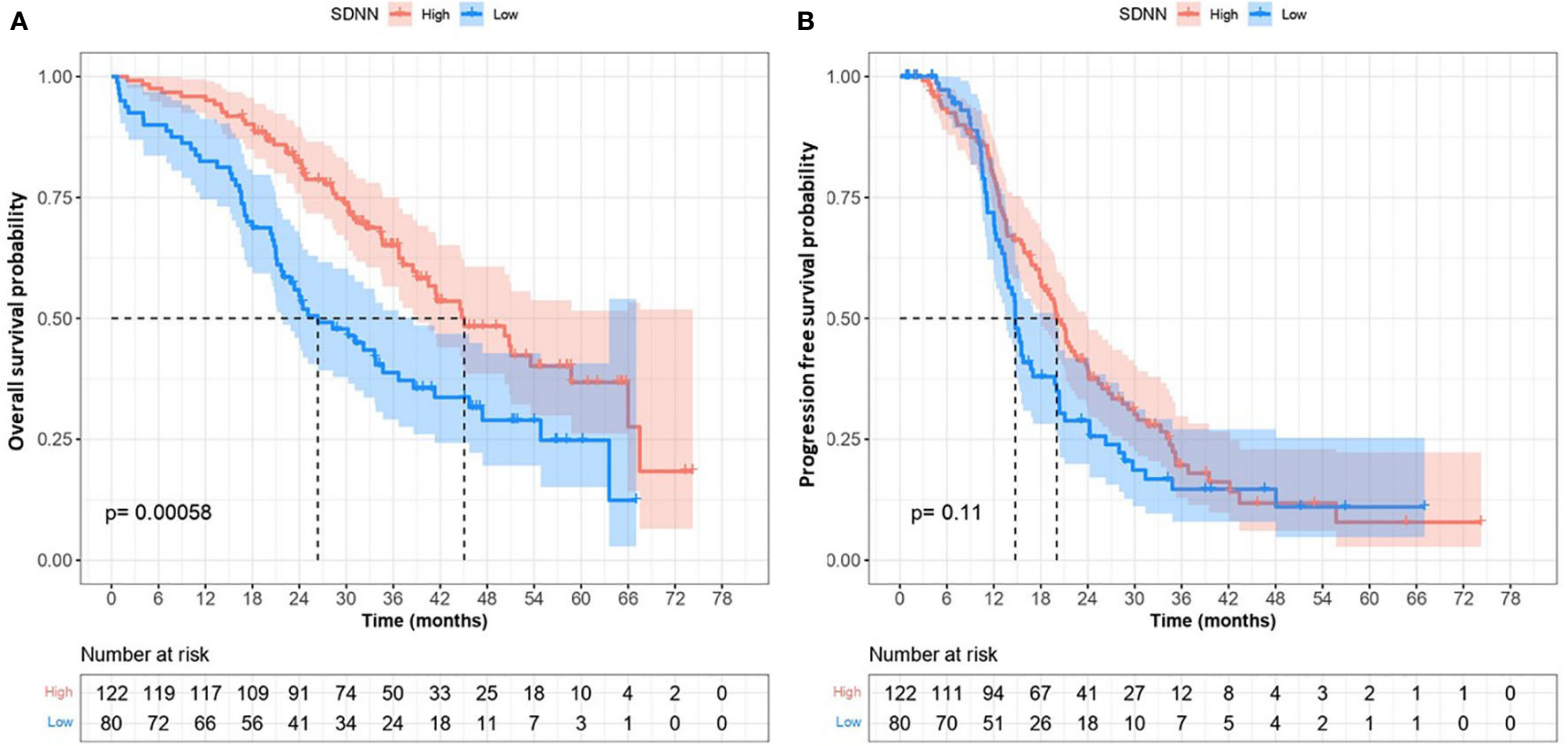
Overall Survival **(A)** and progression free survival **(B)** according to heart rate variability activity (lower versus high).

During the study 110 (54%) died at the time of the analysis, the median OS was 38.6 months (95% CI, 34.4 to 47.4). In univariate analysis we found a statically worse overall survival for patients with low HRV (SDNN <10) compared to high HRV, with median OS of 26.4 vs. 45.1 months, respectively (p<0.001) (**Figure 2A**). Other factors significantly associated with OS in univariate analysis were cancer center, ECOG status, history of diabetes, FIGO stage, R1 surgery (incomplete or no surgery), high NLR and abnormal CA 125 level at the end of first-line treatment (**Table 3**).

**Table 3:** Univariable and multivariable Cox analysis of overall survival.

	Univariable		Multivariable	
	HR [95%CI]	Cox p	HR [95%CI]	Cox p
SDNN low (<10)	1.95 [1.34 - 2.84]	**<0.001**	2.03 [1.35 - 3.06]	**<0.001**
Resection R1	3.41 [2.31 - 5.04]	**<0.001**	2.22 [1.39 - 3.52]	**<0.001**
ECOG >0	3.07 [2.03 - 4.63]	**<0.001**	1.76 [1.09 - 2.86]	**0.019**
CA 125 (>35)	2.7 [1.83 - 3.40]	**<0.001**	1.77 [1.12 - 2.80]	**0.013**
CFB	2.12 [1.43 - 3.14]	**<0.001**	1.60 [1.03 - 2.47]	**0.039**
Diabetes mellitus	1.97 [1.17 - 3.33]	**0.010**	1.23 [0.66 - 2.31]	0.504
FIGO IV	1.84 [1.23 - 2.76]	**0.003**	1.10 [0.68 - 1.77]	0.695
NLR high (> 4)	1.65 [1.11 - 2.44]	**0.013**	1.64 [1.07 - 2.49]	**0.021**
Age ≥ 65	1.50 [0.99 - 2.26]	0.051	1.15 [0.74 - 1.79]	0.523

CFB, Centre François Baclesse Caen France; ECOG, Eastern Cooperative Oncology Group; NLR, Neutrophil-to-lymphocyte ratio; R1, incomplete surgery; SDNN, Standard Deviation of all Normal-to-Normal heartbeat intervals. Bold values are significant value.

#### Multivariate analysis of OS

In multivariate analyses, SDNN remained significantly predictive of OS, with a two-fold higher risk of death among patients with low SDNN compared to those with high SDNN (HR = 2.03, 95%CI = [1.35 - 3.06], p<0.001) (**Table 3**). Other factors significantly associated with OS in the multivariate model included ECOG performance status, R1 surgery, cancer center, high NLR and CA125 levels.

The results were similar when SDNN was analyzed as a continuous parameter, with a higher risk of death observed at lower SDNN values (HR [95%CI] per 1 SD decrease in log-SDNN = 1.46 [1.17 - 1.82], p<0.001 in the multivariate model). It should be noted that if we interpreted age as a continuous variable, it remained non-significant in the multivariate analysis.

#### RMSSD analysis for OS

We confirmed our results using the RMSSD parameter, and 89 patients were considered to have low HRV according to RMSSD. We also found a statistically worse OS for patients with low HRV compared to those with high HRV in the univariate analysis with RMSSD (HR = 2.12, 95%CI= [1.44 - 3.10] (p <0.001) and multivariate analysis (HR = 2.05, 95%CI= [1.37 - 3.06] (p <0.001). Likewise, the results did not change when RMSSD was entered as a continuous parameter in the multivariate model (HR [95%CI] per 1SD decrease in log-RMSSD = 1.44 [95%CI:1.16 - 1.79], p<0.001).

#### HRV and PFS

One hundred and one (75%) patients had disease progression with a median PFS of 18.1 months (95% confidence interval [CI], 15.7 to 20.6). We did not find any association between SDNN and PFS in the univariate analysis with median PFS of 14.7 and 20 months for low SDNN and high SDNN, respectively; HR= 1.31, 95%CI:0.95 - 1.82], p=0.104) (**Figure 2B**). In the multivariate analysis, we did not find any association between HRV and PFS; only R1 resection and CA 125 level above 35UI/ml at the end of the first-line treatment were still significantly associated with PFS. (**Table 4**). The results using RMSSD were similar to those using SDNN in terms of PFS.

**Table 4:** Univariable and multivariable Cox analysis of progression free survival.

	Univariable		Multivariable	
	HR [95%CI]	Cox p	HR [95%CI]	Cox p
SDNN low (<10)	1.31 [0.94 - 1.82]	0.104	1.39 [0.97 - 1.98]	0.066
Resection R1	2.89 [2.07 - 4.04]	**<0.001**	2.08 [1.40 - 3.10]	**<0.001**
ECOG >0	1.55 [1.12 - 2.14]	**<0.001**	1.07 [0.74 - 1.54]	0.711
CA 125 (>35)	2.31 [1.67 - 3.19]	**<0.001**	1.84 [1.28 - 2.63]	**<0.001**
CFB	1.41 [1.02 - 1.94]	**0.038**	1.20 [0.83 - 1.72]	0.333
Diabetes mellitus	1.19 [0.70 - 1.99]	0.516	1.01 [0.57 - 1.81]	0.948
FIGO IV	1.77 [1.22 - 2.57]	**0.003**	1.47 [0.98- 2.18]	0.058
NLR high (> 4)	1.28 [0.92 - 1.80]	0.142	1.36 [0.96 - 1.93]	0.082
Age ≥ 65	0.96 [0.69 - 1.34]	0.817	0.75 [0.52 - 1.07]	0.115

CFB, Centre François Baclesse Caen France; ECOG, Eastern Cooperative Oncology Group; NLR, Neutrophil-to-lymphocyte ratio; R1, incomplete surgery; SDNN, Standard Deviation of all Normal-to-Normal heartbeat intervals. Bold values are significant value.

### Relationship between HRV and CA125/NLR

Among the 80 individuals with low HRV, 52% had abnormal CA125 at the end of the first-line treatment and 47% had abnormal NLR compared to 46% and 35% of individuals with high HRV (p=0.44 and p=0.145, respectively) (**Table 2**). In multivariate model adjusted for age, FIGO stage, ECOG, diabetes and quality of surgery, low HRV (SDNN<10) was associated with higher risk of death (HR=2.15; 95%CI=[1.44-3.20]). The magnitude of association remained similar when further controlling the model for NLR and CA125 levels at the end of the first-line treatment (HR=2.03 [1.35-3.06]), suggesting little mediation by either prognostic factor.

There was no time by HRV interaction with respect to the trajectory of the CA125 levels over time (SDNN, p=0.49; RMSSD, p=0.094) suggesting that the trajectory of CA125 was unrelated to initial HRV.

## Discussion

This study examined the prognostic role of vagal nerve activity in OC. We found that vagal nerve activity indexed by HRV was a strong and independent prognostic factor of overall survival in patients with advanced OC. Patients with low vagal activity had a two-fold higher risk of death than those with high vagal activity, independent of other known prognostic factors.

Our study is one of the largest in this field, focusing on OC. Our results confirm the previous results of studies on other cancer types (colon, lung, breast, etc.) and HRV measures that showed that lower HRV was linked to poorer survival (19, 29). There is no defined cut-off for low vagal activity owing to the high heterogeneity among studies of different tumor types, stages, ECG recordings, and derived HRV parameters (19). In the literature, there is no standard cut-off, which is why we used an ROC curve to determine the optimal cut-off which was 10ms. The cut-off of 20 ms has been mostly used in previous studies on cancer but not for all, and it is often the median that is used (16, 18, 22–25, 30). Some studies used frequency-domain HRV parameters and not time-domain HRV parameters, making comparisons between studies difficult (21). De Cook et al. (16) retrospectively analyzed the norms of vagal nerve activity using SDNN/RMSSD in cancer, which was the only other study to include OC. Among the 58 OC patients, the mean SDNN was 18.95 and the mean RMSSD was 18.17, lower than the mean of other tumor types and lower than the pooled mean across all cancer types (SDNN=21.65 ms, RMSSD=23.19 ms). The median HRV remained higher than that in our study, which may be explained by including patients with earlier cancer stages and younger patients than in the present study, thus a healthier population. It has been shown that HRV decreases with high age and worse stage. Moreover, healthy populations have a higher HRV than patients with cancer (15, 16).

RMSSD has been less explored in cancer patients in the literature, but it is more suitable for the measurement of cardiac vagal nerve activity with less influence from breathing rate (14, 15, 31, 32). Our two parameters of vagal nerve activity, namely SDNN and RMSSD, were strongly associated with each other and with survival, in the same direction and magnitude - the lower the HRV level, the worse was the survival.

In our study, abnormal CA 125 levels at the end of first-line treatment were correlated with OS, independent of HRV level. We did not confirm the results observed in colon cancer patients with low HRV (SDNN ≤20 ms), who had significantly higher CEA levels at 12 months than those with higher HRV, independent of other clinical variables (25). The patients in our study with a high NLR had worse OS in ovarian cancer, as previously reported in the literature (26, 27).

The vagus inhibits inflammation and sympathetic activity (33) which could explain why a high vagal activity predicts better survival. In a study of pancreatic cancer patients, levels of the inflammatory marker C-reactive protein (CRP) mediated the association between HRV and OS (23). We had no sufficient CRP measures available to replicate this finding in patients with OC, but such a mechanism deserves further investigation. Other specific inflammatory markers such as Interleukine-6 or tumor necrosis factor-alpha, may mediate HRV-OS relationships. High NLR, which is another inflammatory biomarker, was significantly associated in multivariate analysis with a worse survival, as observed in literature (34). An inflammatory environment is indeed involved in the progression and survival of OC. However, we found that HRV had a stronger and independent association with OS than NLR in multivariate analysis, which was not in support of a mediation mechanism. This suggests that HRV is a more complex marker than just another correlates of inflammation, because it reflects vagal activity which modulates multiple body systems including immune, cardiovascular and hormonal. These two biomarkers are very relevant because we used complementary parameters already used in clinical practice in the management of OC to compute them (e.g., ECG and blood counts).

The hypothesis of shorter survival when HRV is low is based on a lower parasympathetic/sympathetic balance, which increases tumor aggressiveness. SNS activation leads to the release of catecholamine neuro-effectors (adrenaline and noradrenaline), which modulate gene expression in favor of inflammation (transcription of pro-inflammatory cytokines, inhibition of cellular anti-tumor immune responses, and less programmed cell death in cancer cells), more angiogenesis, epithelial–mesenchymal transition, and tumor invasion (6). The role of the PNS in cancer progression has been less studied, but it may reduce tumor progression by directly counteracting SNS activity. Furthermore, acetylcholine, a major neurotransmitter in the PNS, has anti-inflammatory effects, and the administration of acetylcholinesterase inhibitors attenuates the severity of diseases involving inflammation, decreases the levels of pro-inflammatory cytokines, modulates immune responses and tumor-infiltrating lymphocytes (11, 12, 19, 35).

The time to first recurrence is dictated primarily by treatment effectiveness (1). R1 surgery and high CA 125 near the end of chemotherapy were significantly associated with PFS, as is expected, but not HRV. Vagal activity may not significantly influence the initial therapeutic response, but future studies should investigate this issue further.

Our study has several limitations. ECGs were collected in a retrospective setting, without control over the precise manner of measurement, not systematically practiced, hence we had to exclude a certain number of patients without ECG or with poor ECG quality. ECG collected have been mainly performed just before the diagnosis surgery of ovarian cancer as part of anesthesia consultation, with a median at minus two days, and 97% were performed before the oncological surgery among operated patients. Less frequently ECG were collected at an earlier date, which may indicate another medical condition than OC, notably cardiac disease. However, they were no any significant differences between HRV level groups whether patients had their ECG collected before or after chemotherapy. Moreover ECOG, history of heart disease, history of diabetes mellitus and beta blocker prescription did not differ between the two groups of low and high HRV, hence it is unlikely than major medical conditions biased our results or caused HRV abnormalities other than OC. We used the method validated by Hamilton et al. to measure HRV derived from a short 10 second ECG (28) because it is the duration in normal clinical practice. However, the longer the ECG the more R-R intervals there are and, this may increase the accuracy of the HRV measurement. The gold standard for measuring HRV is often the 5 minutes ECG (14), but several studies focused on ultrashort ECG. Furthermore, a strong correlation between the 5min HRV measurement and the 10second HRV measurement was found, with better accuracy for RMSDD, and do recommended the use of ultrashort ECG (28, 36, 37). In our study, RMSDD and SDNN strongly correlated with each other and similarly predicted prognosis, reinforcing the internal consistency of our measurement. Furthermore, it is important to remember that HRV is not akin to vagus nerve activity and should be interpreted with caution, but it remains a good surrogate marker easy to obtain with the advantage to be more relevant in clinical practice (13, 29). Finally, there was some discrepancy between our two cancer centers, with one center having relatively fitter patients and performing more surgeries with an impact on the mortality rate. One of the centers may have had more referral practices. However, these differences are unlikely to have biased our results, because all analyses were adjusted for the study center as well.

Recent clinical trials have attempted to target PNS and vagal nerve activity as new methods for cancer treatment (5). Beta-adrenergic blockers can block stress-related immunosuppressive pathways of β-adrenergic receptors and could improve tumor responses to immune checkpoint inhibitors. This approach was tested in a phase 1 study of melanoma and showed promising activity without toxicity (38). To counterbalance the activity of the SNS, trials of beta-adrenergic blockade in breast cancer have shown that it can prove to be an effective adjunct to standard treatment with few adverse effects (39). In a small retrospective study of OC patients, beta-blocker use was associated with better PFS and OS, with a 54% reduced chance of death compared with non-users of beta-blockers (40). In a review of supportive therapy modalities in cancer patients, exercise, music therapy, and relaxation techniques (yoga, Tai-Chi Qigong, and breathing techniques) were shown to have the potential to enhance vagal nerve function. These techniques can increase parasympathetic autonomic activity by improving HRV. These were small heterogeneous studies that were not always randomized; however, their results deserve further exploration. In this context, HRV analysis appears to be an easily applicable and a safe method for evaluating cancer-related autonomic dysfunction (10). Larkey et al., in a mind-body and psychosocial intervention study, found that changes in HRV parameters were associated with improved ANS balance. HRV can serve as a marker of underlying changes in stress responses among cancer survivors (41). Finally, a small non-randomized but matched-controlled pilot study compared three months of vagal breathing *via* HRV biofeedback and chemotherapy, to controls who received only chemotherapy and matched for tumor type and stage, chemotherapy line, and baseline CEA levels. Over the three months, patients receiving the combined treatment showed far greater reductions in CEA levels than the controls (42).

Knowing the HRV status of patients may have direct application in this promising approach to cancer neuroscience and neuromodulation therapeutics, whether pharmacologically or not.

## Conclusions

Heart rate variability of advanced ovarian cancer is strongly and independently associated with OS. Patients with low vagal activity had a lower chance of survival. If our results are confirmed in a large longitudinal study, therapies aimed at stimulating/reactivating the functioning of the parasympathetic vagal nervous system may be explored. Heart rate variability could be used to monitor such therapies and to estimate prognosis in OC.

## Data availability statement

The raw data supporting the conclusions of this article will be made available by the authors, without undue reservation.

## Ethics statement

Informed consent was obtained from all the participants. This study categories in “Research not involving Human participants” of the French Public Health Code, articles 53 of the Data Protection Act, and respects the MR04 standards and have been performed in accordance with the Declaration of Helsinki. This study was approved by the institutional review board of the Cancer Center, François Baclesse, Caen, France. It was registered in the French Health Data Hub under reference N° F20201023132151. Promoter: The cancer center François Baclesse, Caen, France, complies with the regulations in force, in particular, the rights of persons subject to processing within the meaning of EU Regulation No. 2016/679 on data protection (“RGPD”).

## Author contributions

FC, conceptualization, data curation, and writing - original draft. JF, data curation, writing - review and editing. SL and JL, formal analysis, and writing - original draft. SB, CA, NP, and EC, Writing - review and editing. YG, conceptualization, formal analysis, writing - review and editing. FJ, conceptualization, formal analysis, and writing - original draft. All authors contributed to the article and approved the submitted version.

## Funding

The Center François Baclesse, Caen, France paid for the publication fee of the article.

## Conflict of interest

FJ reports participating in advisory boards with AstraZeneca, GSK, Clovis, MSD and seagen, reports consulting fees from GSK, and support for attending meetings from GSK, Astrazeneca and MSD. YG was a chair in psycho-oncology during 2017-2019 from the French National Cancer Institute.

The remaining authors declare that the research was conducted in the absence of any commercial or financial relationships that could be construed as a potential conflict of interest.

## Publisher’s note

All claims expressed in this article are solely those of the authors and do not necessarily represent those of their affiliated organizations, or those of the publisher, the editors and the reviewers. Any product that may be evaluated in this article, or claim that may be made by its manufacturer, is not guaranteed or endorsed by the publisher.
